# The right touch: Stroking of CT-innervated skin promotes vocal emotion processing

**DOI:** 10.3758/s13415-017-0537-5

**Published:** 2017-09-20

**Authors:** Annett Schirmer, Thomas C. Gunter

**Affiliations:** 1 Department of Psychology, The Chinese University of Hong Kong, Shatin, Hong Kong; 2 Department of Neuropsychology, Max Planck Institute for Human Cognitive and Brain Sciences, Leipzig, Germany

**Keywords:** EEG, ERP, Nonverbal, Emotion recognition, Somatosensory

## Abstract

Research has revealed a special mechanoreceptor, called C-tactile (CT) afferent, that is situated in hairy skin and that seems relevant for the processing of social touch. We pursued a possible role of this receptor in the perception of other social signals such as a person’s voice. Participants completed three sessions in which they heard surprised and neutral vocal and nonvocal sounds and detected rare sound repetitions. In a given session, participants received no touch or soft brushstrokes to the arm (CT innervated) or palm (CT free). Event-related potentials elicited to sounds revealed that stroking to the arm facilitated the integration of vocal and emotional information. The late positive potential was greater for surprised vocal relative to neutral vocal and nonvocal sounds, and this effect was greater for arm touch relative to both palm touch and no touch. Together, these results indicate that stroking to the arm facilitates the allocation of processing resources to emotional voices, thus supporting the possibility that CT stimulation benefits social perception cross-modally.

**Electronic supplementary material:**

The online version of this article (10.3758/s13415-017-0537-5) contains supplementary material, which is available to authorized users.

We, like many other animals, engage in friendly physical contact. Initially, this phenomenon was attributed to the hygienic needs of our furry ancestors and considered of little current value. However, the discovery that nonhuman primates groom more than is hygienically necessary changed this view (Dunbar, 2010). It highlighted that, besides keeping clean, touch has additional functions that could be preserved in humans. Here, we pursued this possibility by exploring the effect of touch on the neural correlates of voice perception.

Behavioral work has fostered the idea that touch promotes positive affect and facilitates social bonding (Dunbar, 2010; Gallace & Spence, 2010; Suvilehto, Glerean, Dunbar, Hari, & Nummenmaa, 2015). Neuroscience, in turn, has revealed a tactile system that could underpin such effects (Iggo, 1960; Nordin, 1990). This system comprises C-tactile (CT) afferents, which differ from Aβ mechanoreceptors in a number of ways. For example, they are unmyelinated, occur in nonglabrous skin only, and seem tuned to the physical characteristics of human touch. Specifically, they are maximally excited by low-pressure physical contact with typical body temperature (Ackerley et al., 2014) and a stroking speed of 1 to 10 cm per second (Löken, Wessberg, Morrison, McGlone, & Olausson, 2009). Moreover, their firing rates linearly predict subjective pleasure (Ackerley et al., 2014; Löken et al., 2009; Olausson et al., 2002; Olausson, Wessberg, Morrison, McGlone, & Vallbo, 2010). Looking at their projections, CT afferents behave similarly to other C fibers relevant for interoception and pain (Björnsdotter, Morrison, & Olausson, 2010). They have a slow conduction velocity and send fibers to the insula by-passing primary somatosensory cortex (Kaiser et al., 2015; Olausson et al., 2002). Thus, patients without Aβ afferents but intact CT afferents may experience a vague sense of pleasure from the stroking of CT-innervated skin despite being unable to properly discriminate the tactile sensation (Olausson et al., 2002).

Based on these findings, CT afferents have been proposed to underpin the affective and rewarding qualities of touch in social interactions (McGlone, Wessberg, & Olausson, 2014; Olausson et al., 2008; Olausson et al., 2002). Additionally, and this possibility was tested here, they may promote the perception of other social signals (e.g., voices). This possibility derives from the fact that CT projections reach the posterior superior temporal sulcus (Bennett, Bolling, Anderson, Pelphrey, & Kaiser, 2014; Kaiser et al., 2015), a known social processing hub that prefers social over nonsocial information and that integrates this information across modalities (Schirmer & Adolphs, 2017; Watson, Latinus, Charest, Crabbe, & Belin, 2014).

To explore CT effects on social processing, we recorded the electroencephalogram (EEG) while participants listened to emotional and neutral vocal and nonvocal sounds on the backdrop of CT-appropriate stroking on the arm, comparable stroking on the palm, or no stroking. We expected CT touch to modulate two event-related potential (ERP) components previously associated with emotion recognition and social perception. One component was an early positive deflection peaking around 200 ms following sound onset (P2), with greater amplitudes for emotional as compared to neutral (Jiang & Pell, 2015; Paulmann & Pell, 2010; Schirmer, Chen, Ching, Tan, & Hong, 2013; Schirmer & Gunter, 2017) and social as compared to nonsocial sounds (Charest et al., 2009; Schirmer & Gunter, 2017). The other component was a late positive potential (LPP) known for its sensitivity to a range of modality-unspecific stimulus (e.g., salience) and task (e.g., relevance) characteristics. For example, emotionality (Amrhein, Mühlberger, Pauli, & Wiedemann, 2004) and humanness (Schindler & Kissler, 2016) robustly increase the LPP. Additionally, the integration of different stimulus dimensions and of stimulus with contextual information is linked to this component (Diéguez-Risco, Aguado, Albert, & Hinojosa, 2013; Schirmer & Gunter, 2017).

In line with established evidence, we predicted larger P2 and LPP amplitudes for vocal as compared to nonvocal and emotional as compared to neutral stimuli. Moreover, we hypothesized emotion effects to be larger for vocal relative to nonvocal sounds, especially for later integrative processing in the LPP (Schirmer & Gunter, 2017). Critically, enhanced responses to vocal-emotional sounds should be amplified further by touch to CT-innervated skin if such touch interfaces with and promotes social perception.

## Method

### Participants

Eighteen women were recruited for this study. We focused on female participants because of established sex differences in nonverbal sensitivity (e.g., Schirmer & Gunter, 2017) and touch (Fisher, Rytting, & Heslin, 1976) for which exploration would have required a more complex design crossing participant and experimenter sex (18 participants in 4 groups—ff, fm, mf, mm—with 3 recording sessions = 216 recording sessions). No participants were excluded from data analysis. The number of participants was set a priori based on session counterbalancing and had to be a multiple of 6. All participants reported normal hearing and an absence of neurological impairments. All were right-handed as assessed with the Edinburgh Handedness Inventory and were on average 25 (*SD* = 2.6) years old. This research was conducted in accordance with the Declaration of Helsinki. All participants signed informed consent at the beginning of the experiment.

### Stimulus materials

Vocalizations were taken from a previous study for which they were selected and normed (Schirmer & Gunter, 2017). In short, 33 speakers pronounced “Ah” neutrally as well as with anger, disgust, fear, happiness, sadness, and surprise. Recordings were made by the present investigators as well as Belin, Fillion-Bilodeau, and Gosselin (2008) in a soundproof chamber and digitized at 16 bits/44.1 kHz.

Thirty participants (12 male, 18 female, mean age = 22.07 years), not contributing to the main experiment, classified each vocalization as angry, disgusted, fearful, happy, sad, surprised, neutral or “other” in case none of the aforementioned options seemed adequate. For sounds not categorized as neutral, the participants were prompted to rate emotion intensity and arousal on two 5-point scales ranging from 1 (*very weak*) to 5 (*very strong*).

For this project, we selected the 27 best recognized surprise expressions (mean accuracy = 73.70%, *SD* = 20.47; mean intensity = 3.29, *SD* = 0.459; mean arousal = 3.26, *SD* = 0.401) as well as 27 matching neutral sounds (mean accuracy = 76.91%, *SD* = 16.77). We decided to use one rather than multiple emotions because we wanted to control stimulus variation/homogeneity/probability between the neutral and the emotion condition. Moreover, we selected surprise because of its good recognition and high arousal value. All selected sounds were normalized at the same root-mean-square value and subjected to spectral rotation (http://www.phon.ucl.ac.uk/resource/software.php), resulting in a comparable set of nonvocal sounds (Warren et al., 2006). As such, these sounds retained some affective content but were perceived as distinctly nonhuman. (For a more detailed acoustic and perceptual analysis, see Schirmer & Gunter, 2017.) Exemplary sounds may be downloaded here (https://osf.io/5n8md/).

**Fig. 1:**
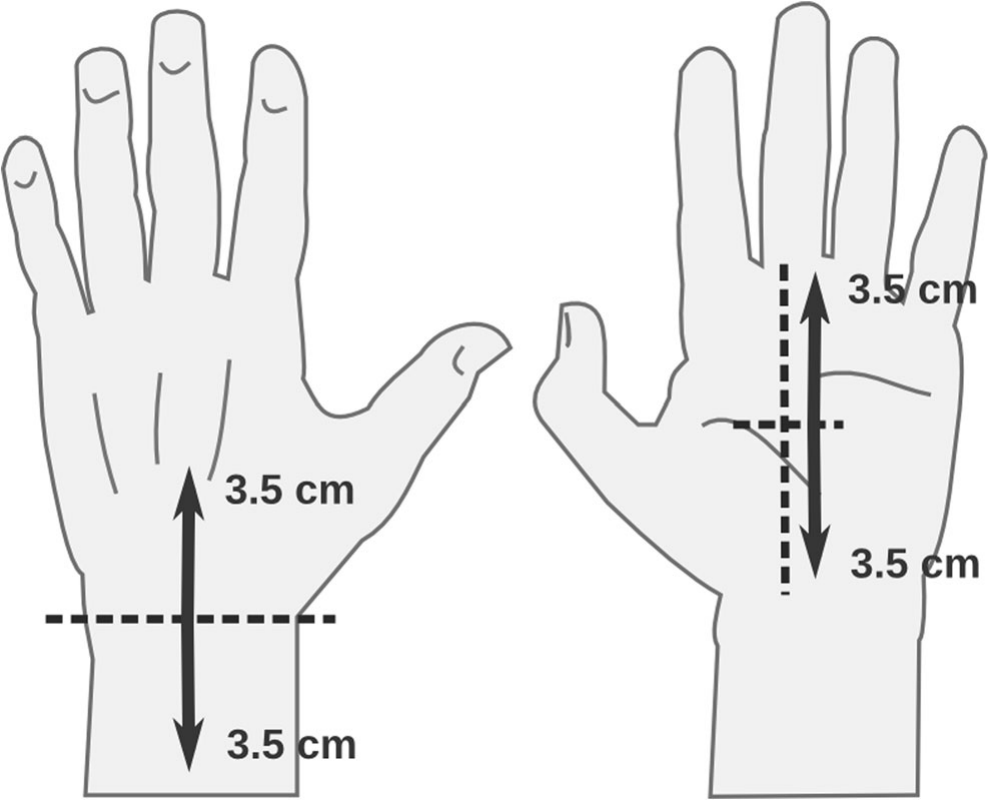
Touch preparation. Stroking areas for arm (left) and palm (right) are indicated by dark arrows

The touch stimulation was applied via a hand-held soft cosmetic brush, 3-cm in diameter, on short sections of the arm and palm. For the arm section, the experimenter first identified the transition between hand and wrist and then used a makeup pen to draw a 3.5-cm upward and downward boundary. For the palm section, the experimenter identified the palm midpoint and used the makeup pen to draw a 3.5-cm upward and downward boundary (see Fig. 1). Stroking was done by the experimenter at a speed of 3.5 cm per second. A repetitive fading tone was delivered to the experimenter over headphones. The tone was played every 2 seconds to facilitate accurate and constant stroke timing.

### Procedure

Participants were tested individually on 3 days, separated by a week or longer. On these days, they interacted with the same female experimenter, who prepared them for the task and subsequently applied the touch condition. On each testing day, participants first underwent a standard setup procedure for the EEG recording. The EEG was recorded using 59 Ag/AgCl electrodes, which were located according to sites defined in the extended 10–20 system of the American Clinical Neurophysiology Society (2006). Individual electrodes were attached above and below the right eye and at the outer canthus of each eye to measure eye movements. One electrode was attached to the nose for data referencing. The nose was chosen as to facilitate comparisons with related research (Schirmer & Gunter, 2017) and the exploration of a possible auditory cortex involvement (Näätänen, Paavilainen, Rinne, & Alho, 2007). Electrode impedance was below 5 KΩ. The data was recorded at 500 Hz with a BrainAmp EEG system. Only an anti-aliasing filter was applied during data acquisition (i.e., sinc filter with a half-power cutoff at half the sampling rate).

Following the EEG setup, participants were prepared for both arm and palm touch—irrespective of whether they actually received this touch on that day. The experimenter used an alcohol swab to clean an ~8-cm area on both arm and palm and proceeded to mark the touch sections as described under “Stimulus Materials.” Subsequently, participants moved into the experimental chamber and sat down in front of a computer screen that was framed by two speakers. Next to them sat the female experimenter. A curtain separated both and allowed the participant to place his or her left arm out of sight, on a board accessible to the experimenter. The experimenter sat next to the participant during all three sessions, irrespective of whether she applied strokes.

Instructions presented on the screen informed participants that they would hear a sequence of sounds and that their task was to press the button on a button box (Dimension 16 × 8 × 1.5 cm; C&K digitast SERU switch/gray SER button was positioned at the lower middle position 8:2.5 cm) placed at the end of the right armrest using their right hand any time a sound was the same as the one before (one-back task). The task comprised three blocks in which sounds (i.e., 27 neutral/vocal, 27 surprised/vocal, 27 neutral/nonvocal, and 27 surprised/nonvocal) were played once in random order, with the constraint that a vocal stimulus and its spectral rotation (i.e., nonvocal counterpart) appeared in separate block halves as to avoid the emergence of potential acoustic associations. Additionally, 22 sounds were randomly selected for repetition in each block, which served to engage participants with the auditory material without highlighting the nature of the sounds and without necessitating a confounding motor response on nonrepeated, experimental trials. As such, each of the three blocks comprised 108 nonrepeated and 22 repeated trials. Across the experiment, a total of 324 sounds not requiring a motor response were designated for the EEG analysis, whereas 66 sounds requiring a motor response were designated for behavior analysis.

Each task trial started with a white fixation cross centered on a black background. After 500 ms, a sound played (average duration = 506 ms; *SD* = 25 ms) and the fixation cross remained for 1,000 ms after which it disappeared. An empty intertrial interval had a random duration between 2,000 and 4,000 ms.

In a given session, participants completed the task either with continuous stroking to the arm, the palm, or no stroking. Although CT afferents fatigue after repeated stroking, they do continue to fire (Nordin, 1990), and subjective experiences even after 50 minutes of stimulation are not negative (Triscoli, Ackerley, & Sailer, 2014). The order of touch conditions across the three sessions was fully counterbalanced. A session lasted about 30 minutes and included short breaks between blocks.

Before and after completing the task, participants indicated their current mood on a 5-point scale ranging from −2 (*very negative*) to +2 (*very positive*). Then they indicated how aroused they feel on a 5-point scale ranging from 0 to 4. Last, and only at the end of the task, they rated touch pleasantness on a 5-point scale ranging from −2 (*very unpleasant*) to +2 (*very pleasant*) if they had been stroked by the experimenter.

### Data analysis

EEG data were processed with EEGLAB (Delorme & Makeig, 2004). The recordings were subjected to low-pass and high-pass filtering with a half-power cutoff at 30 and 0.1 Hz, respectively. The transition band was 7.5 Hz for the low-pass filter (−6 dB/octave; 221 pts) and 0.1 Hz for the high-pass filter (−6 dB/octave; 1,6501 pts). The continuous data were epoched using a 1,000-ms prestimulus window and a 1,000-ms poststimulus window. The resulting epochs were visually scanned for nontypical artifacts caused by drifts or muscle movements. Epochs containing such artifacts were removed. After the application of a 1 Hz high-pass filter, the data were subjected to an independent component analysis (Onton, Westerfield, Townsend, & Makeig, 2006) and the component structure resulting from this analysis was applied to the original epoched data set with the 30 to 0.1 Hz filter setting (Winkler, Debener, Müller, & Tangermann, 2015). Components reflecting typical artifacts (i.e., horizontal and vertical eye movements and eye blinks) were removed, and the data back-projected from component space into EEG channels space, re-epoched and baseline-corrected using a 200-ms prestimulus window and a 1,000-ms poststimulus window. The resulting epochs were again scanned visually for residual artifacts and affected epochs dropped from further analysis. ERPs were derived by averaging the remaining epochs for each condition and participant. A minimum of 58 trials and an average of 75 trials per condition entered statistical analysis.

The analysis windows for the two target ERP components was identified based on visual inspection of the average ERP waveforms as well as previous evidence (i.e., P2: 150–350 ms; LPP: 400–950 ms; Schirmer & Gunter, 2017). Mean voltages from within both windows were subjected to separate ANOVAs, with Voiceness (vocal, nonvocal), Emotion (surprised, neutral), Touch (arm, palm, no), Hemisphere (left, right), and Region (anterior, central, posterior) as repeated-measures factors. The factors Hemisphere and Region comprised average voltages computed across the following subgroups of electrodes: anterior left, Fp1, AF7, AF3, F5, F3, F1; anterior right, Fp2, AF8, AF4, F6, F4, F2; central left, FC3, FC1, C3, C1, CP3, CP1; central right, FC4, FC2, C4, C2, CP4, CP2; posterior left, P5, P3, P1, PO7, PO3, O1; posterior right, P6, P4, P2, PO8, PO4, O2. This selection of electrodes was based on previous research on emotional voice perception (Schirmer & Gunter, 2017) and ensured that the tested subgroups contained an equal number of electrodes while providing a broad scalp coverage that allowed the assessment of topographical effects. Our significance threshold was *p* = .05. We only report main effects and interactions involving the three experimental factors and only if the simple effects in follow-up analyses were at least marginally significant (*p* < .1). We report the generalized eta squared (η_G_^2^) as an effect-size measure for all effects and, if applicable, Greenhouse–Geisser corrected *p* values (p_GG_). An analysis of data referenced to the average of all electrodes is provided in the Supplementary Materials.

## Results

### Behavior

**Fig. 2:**
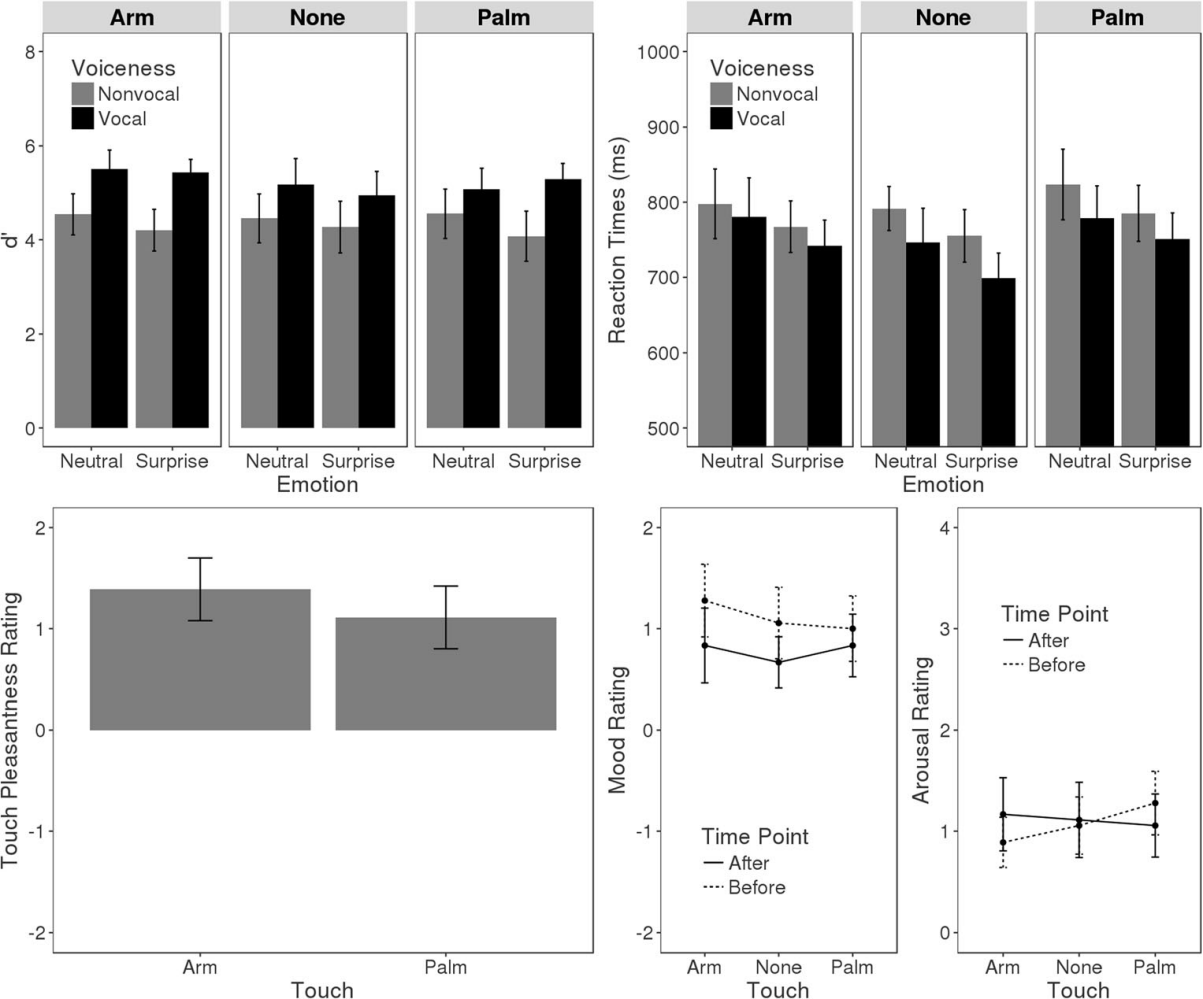
Behavioral results. The upper row presents *d*-primes and reaction times for the target detection task as a function of Touch, Voiceness, and Emotion. The lower row presents the rating scores from the preexperimental and postexperimental rating. Error bars indicate the 95% confidence interval

We analyzed performance in the target detection task to determine possible effects of touch. To this end, reaction times to correctly detected targets as well as *d*-prime scores derived by subtracting the normalized probability of false alarms from the normalized probability of hits were subjected to separate ANOVAs, with Voiceness (vocal, nonvocal), Emotion (surprised, neutral), and Touch (arm, palm, none) as repeated-measures factors. Results are illustrated in Fig. 2.

Correct response times showed main effects of Voiceness, *F*(1, 17) = 18.45, *p* < .001, η_G_^2^ = .018, and Emotion, *F*(1, 17) = 20.67, *p* < .001, η_G_^2^ = .017. Response times were shorter for vocal relative to nonvocal and for surprised relative to neutral sounds. *D*-prime analysis revealed a Voiceness main effect, *F*(1, 17) = 40.94, *p* < .001, η_G_^2^ = .164, indicating that responses were more accurate to vocal than to nonvocal sounds. The interaction of Emotion and Voiceness showed as a tendency, *F*(1, 17) = 3.51, *p* = .078, η_G_^2^ = .005, but follow-up test for vocal (*p* > .250) and nonvocal sounds (*p* = .102) were nonsignificant. The effect of Touch on response times and *d*-primes was nonsignificant (*p*s > .1)

A one-way ANOVA conducted on touch pleasantness ratings revealed no effect (*p* > .25). A two-way ANOVA on mood scores from before and after each session was conducted, with Touch and Rating Time Point (before, after) as repeated-measures factors. This revealed a main effect of Rating Time Point, *F*(1, 17) = 4.78, *p* = .043, η_G_^2^ = .038, indicating that participants felt more positive before than after the experiment. All other effects were nonsignificant (*p* > .25). An ANOVA on arousal scores was nonsignificant (*p*s > .213).

### ERP

**Fig. 3:**
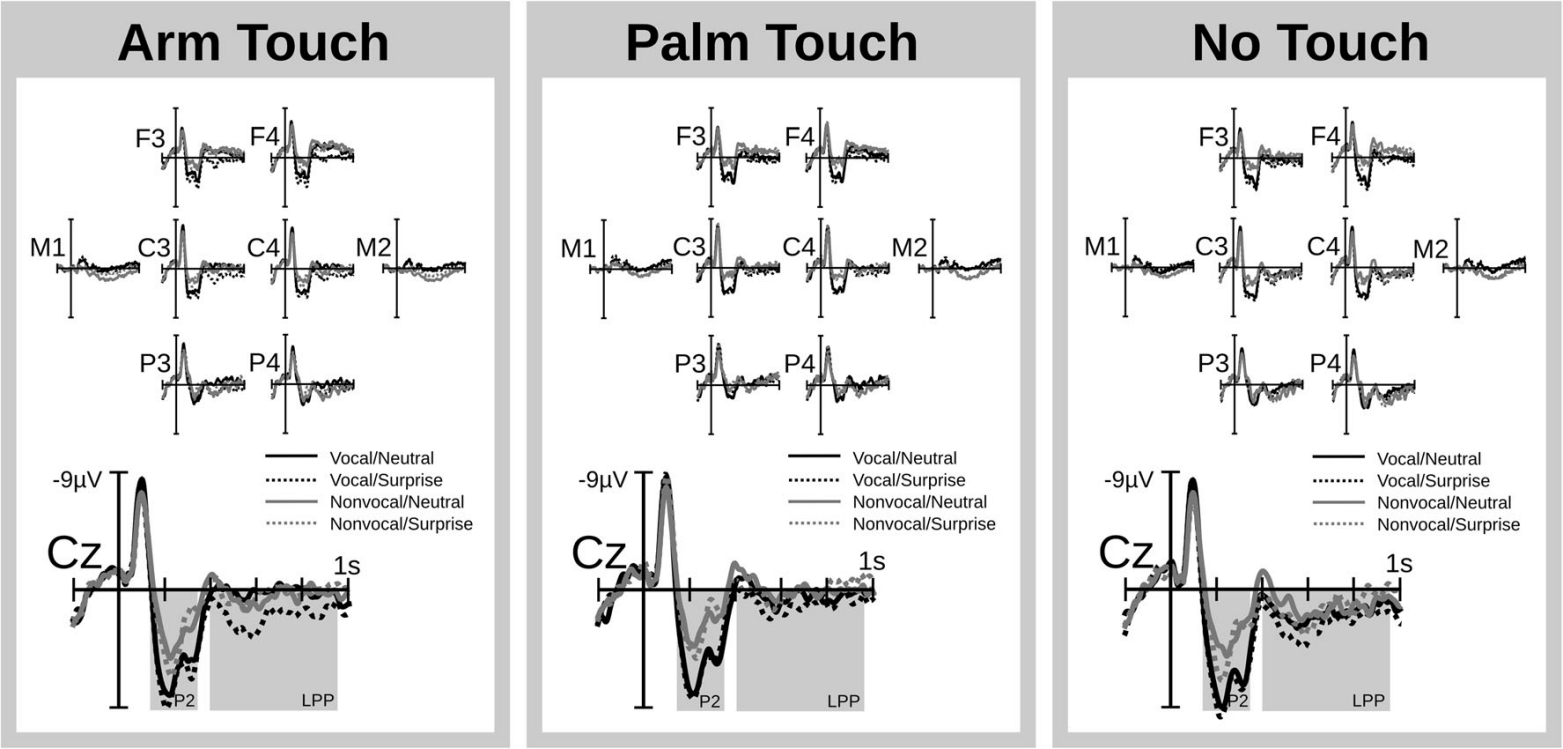
ERP traces. ERPs time locked to sound onset show voiceness and emotion effects that are independent of each other in the P2 and that interact with each other in the LPP. Illustrated are a subset of frontal (F), central (C), and parietal (P) recordings as well as mastoid recordings (M) from the left (uneven digit) and right (even digit) hemisphere. A vertex recording (Cz) is enlarged for better visibility. The interaction of voiceness and emotion in the LPP is stronger for the arm-touch than for palm-touch and no-touch conditions. The two statistical windows (P2, LPP) are shaded in gray

**Fig. 4:**
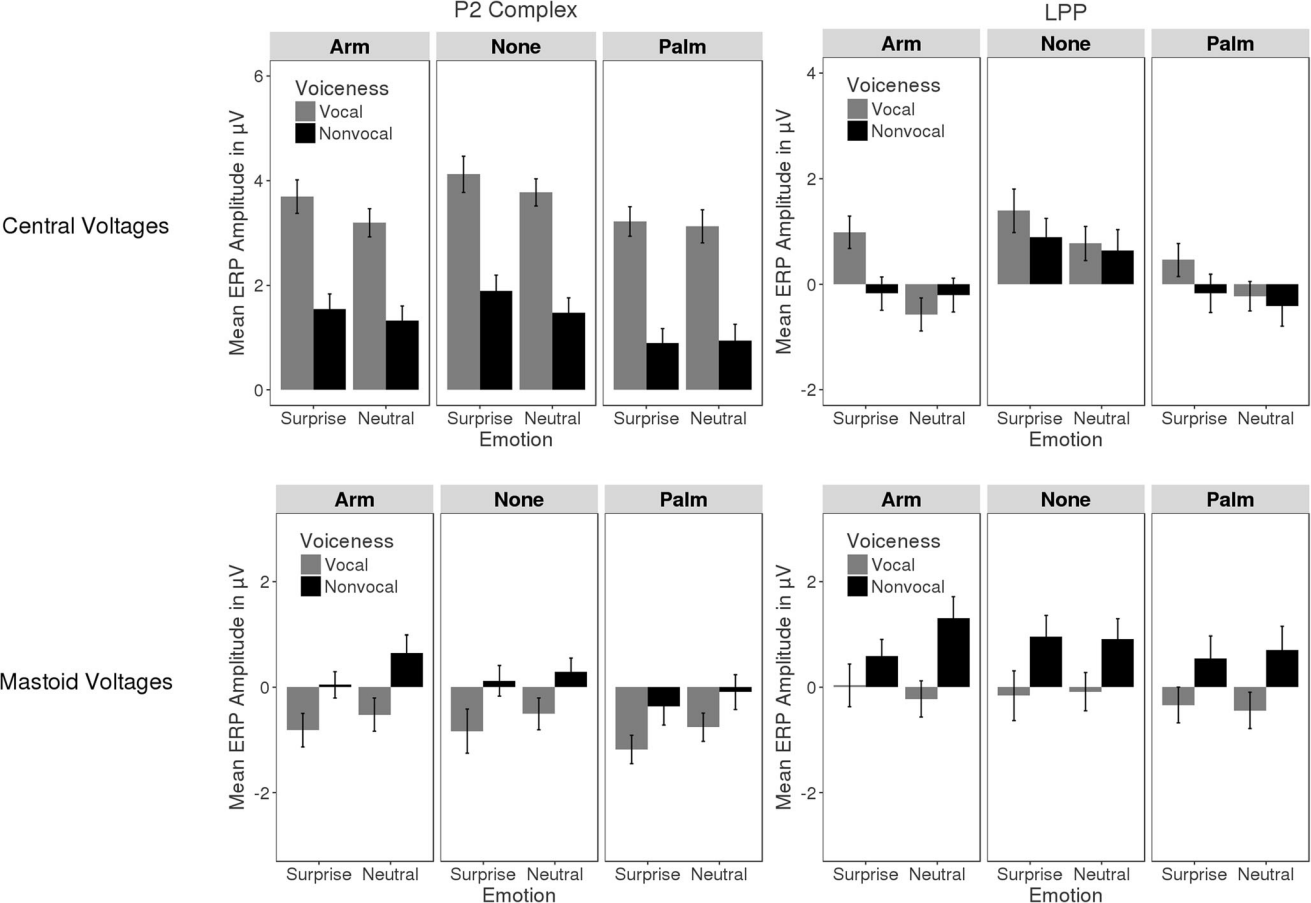
Bar graph of P2 (left) and LPP (right) mean voltages for the different experimental conditions computed across the left and right central region (top row) and the mastoids (bottom). Voiceness effects are most evident in the P2 with greater amplitude for vocal as compared with nonvocal sounds over central and the opposite over mastoid electrodes. Touch effects are most evident in the LPP where they interact with voiceness and emotion. Error bars reflect the 95% confidence interval

**Fig. 5:**
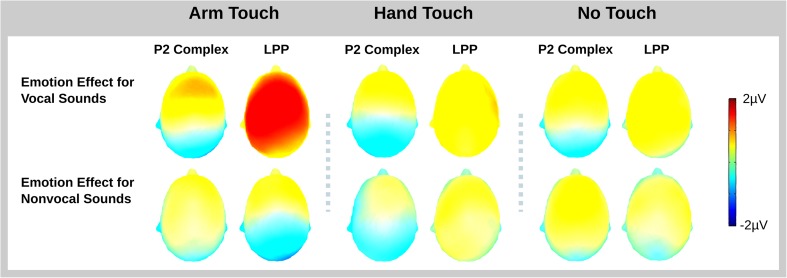
Topography of the voiceness by emotion interaction in the ERP. We plotted mean voltage differences computed by subtracting neutral from surprised sounds for vocal (top row) and nonvocal (bottom row) conditions within the two statistical windows. An interaction between voiceness and emotion was significantly stronger for the LPP in the arm-touch compared to the palm-touch and no-touch conditions. (Color figure online)

ERP time courses are illustrated in Fig. 3. Figure 4 presents a bar graph of mean voltages for the two analysis windows and the different experimental conditions. The topography of the interaction of Emotion, Voiceness, and Touch is illustrated in Fig. 5.

#### P2 complex

Visual inspection revealed a positive complex between 150 and 350 ms that was characterized by a large positive deflection with a small negative deflection superimposed. This pattern points to the presence of multiple components apart from a P2 including an N2, a P3 and/or a sound offset potential. For ease of reference, these patterns will be subsumed under the term P2 complex. Mean voltages of the P2 complex were subjected to an ANOVA, as described in the Method section. The results entailed an effect of Touch, *F*(2, 34) = 3.91, *p* = .03, η_G_^2^ = .014, marking greater P2 amplitudes for no touch relative to palm touch, *F*(1, 17) = 8.84, p = .008, η_G_^2^ = .022. Arm touch and palm touch (*p* = .149) as well as arm touch and no touch (*p* > .250) did not differ. All other effects involving Touch were nonsignificant (*p*s > .1).

The Voiceness main effect, *F*(1, 17) = 4.85, *p* < .001, η_G_^2^ = .074, was significant, as were the interactions of Voiceness and Region, *F*(2, 34) = 6.14, p_GG_ < .001, η_G_^2^ = .026, and Voiceness, Region, and Hemisphere, *F*(2, 34) = 7.93, p_GG_ = .007, η_G_^2^ = .0005. The latter effect was pursued for each level of Region. At anterior electrodes, the Voiceness × Hemisphere interaction, *F*(1, 17) = 6.45, *p* = .021, η_G_^2^ = .003, indicated that an increase in P2 amplitudes for vocal as compared to nonvocal sounds was larger over the right, *F*(1, 17) = 160.16, *p* < .0001, η_G_^2^ = .268, as compared to the left, *F*(1, 17) = 99.42, *p* < .001, η_G_^2^ = .258, hemisphere. At central electrodes, Voiceness enhanced P2 amplitudes irrespective of laterality, F(1, 17) = 77.64, p < .0001, η_G_^2^ = .141. At posterior electrodes, Voiceness interacted again with Hemisphere, *F*(1, 17) = 4.91, *p* = .04, η_G_^2^ = .0003, but follow-up analyses were nonsignificant (*p*s > .216).

Emotion became relevant in interaction with Region, *F*(2, 34) = 36.9, p_GG_ < .0001, η_G_^2^ = .0041, with Region and Hemisphere, *F*(2, 34) = 8.25, p_GG_ = .002, η_G_^2^ = .00007, as well as with Voiceness and Region, *F*(2, 34) = 4.62, p_GG_ = .039, η_G_^2^ = .0006. Follow-up analyses for each region revealed that the Emotion × Voiceness interaction merely approached significance anteriorly, *F*(1, 17) = 3.59, *p* = .075, η_G_^2^ = .002, and was nonsignificant centrally and posteriorly (*p*s > .250). Similarly, the Emotion × Hemisphere interaction merely approached significance posteriorly, *F*(1, 17) = 3.38, *p* = .083, η_G_^2^ = .00005, and was nonsignificant centrally and anteriorly (*p*s > .139). Therefore, these effects were not pursued further. Instead, we focused on the Emotion main effect for each level of Region, which indicated that, anteriorly, P2 voltages were more positive for surprise than neutral sounds, *F*(1, 17) = 28.62, *p* < .0001, η_G_^2^ = .0162*.* Centrally, a similar effect was nonsignificant, *F*(1, 17) = 4.26, *p* = .055, η_G_^2^ = .0023, and posteriorly the effect reversed polarity, *F*(1, 17) = 6.11, *p* = .024, η_G_^2^ = .0031.

#### Late positive potential

Mean voltages between 400 and 950 ms were subjected to an ANOVA as described in the Method section. Whereas Emotion produced a significant main effect, *F*(1, 17) = 18.85, *p* < .001, η_G_^2^ = .005, the effects of Touch, *F*(2, 34) = 2.66, *p* = .085, η_G_^2^ = .019, and Voiceness were nonsignificant (*p* > .25). However, all three factors participated in a number of interactions, including Emotion and Voiceness, *F*(1, 17) = 5.17, *p* = .036, η_G_^2^ = .004; Emotion and Region, *F*(2, 34) = 5.67, p_GG_ = .024, η_G_^2^ = .0009; Voiceness and Region, *F*(2, 34) = 48.66, p_GG_ < .001, η_G_^2^ = .017; Emotion, Voiceness, and Touch, *F*(2, 34) = 3.33, *p* = .047, η_G_^2^ = .001; as well as Emotion, Voiceness, Touch, and Region, *F*(4, 68) = 2.61, *p* = .043, η_G_^2^ = .0004.

We explored the latter effect for each level of Region. At anterior electrodes, the interaction of Emotion, Voice, and Touch was nonsignificant (*p* > .250). Instead the Emotion × Voiceness interaction, *F*(1, 17) = 4.9, *p* = .041, η_G_^2^ = .002, indicated that across touch conditions, surprised sounds increased the LPP relative to neutral sounds when they were vocal, *F*(1, 17) = 28.4, *p* < .001, η_G_^2^ = .027, and to a smaller degree when they were nonvocal, *F*(1, 17) = 6.83, *p* =.018, η_G_^2^ = .005. At central and posterior electrodes, the interaction of Emotion, Voiceness and Touch was significant: central, *F*(2, 34) = 3.4, *p* = .045, η_G_^2^ = .002; posterior, *F*(2, 34) = 4.49, *p* = .018, η_G_^2^ = .002. To probe differences between the two touch conditions, specifically, we repeated these analyses excluding the no-touch condition. In other words, we conducted an Emotion × Voiceness × Touch ANOVA in which Touch had two levels only (arm, palm). A significant three-way interaction at posterior electrodes, *F*(1, 17) = 5.82, *p* = .027, η_G_^2^ = .0036, pointed to a differentiation between arm touch and palm touch. The three-way interaction was nonsignificant at central, *F*(1, 17) = 3.62, *p* = .074, η_G_^2^ = .0032, and anterior electrodes (*p* > .25).

We pursued the interaction of Emotion and Voiceness for arm touch*,* palm touch, and no touch over central and posterior sites. For arm touch, the interaction was significant: central, *F*(1, 17) = 11.9, *p* = .003, η_G_^2^ = .024; posterior, *F*(1, 17) = 11.64, *p* = .003, η_G_^2^ = .028, indicating that the LPP was larger for surprised as compared to neutral vocal but not nonvocal sounds: central, *F*(1, 17) = 29.87, *p* < .001, η_G_^2^ = .091; posterior, *F*(1, 17) = 6.95, *p* = .017, η_G_^2^ = .033. Moreover, for nonvocal sounds, this Emotion effect was absent over central (*p* > .25) and reversed polarity over posterior regions, *F*(1, 17) = 5.81, *p* = .027, η_G_^2^ = .023. During palm touch and no touch, the interaction of Emotion and Voiceness was nonsignificant (*p*s > .193), as was the Emotion main effect: central palm, *F*(1, 17) = 4.27, *p* = .054, η_G_^2^ = .009; central none, *F*(1, 17) = 3.91, *p* =.065, η_G_^2^ = .005; posterior palm/none (*p*s > .250).

#### Mastoid analysis

A polarity inversion of aforementioned auditory processing effects over the mastoid electrodes would be compatible with a source in auditory cortex and in line with prior evidence for a role of sensory regions in both early (Schirmer & Escoffier, 2010) and late emotion ERPs (Liu, Huang, McGinnis, Keil, & Ding, 2012). To probe this possibility, we subjected mastoid recordings of P2 and LPP time windows to separate analyses with Touch, Emotion, Voiceness and Hemisphere as repeated measures factors.

Analysis in the P2 window revealed main effects of Emotion, *F*(1, 17) = 24.87, *p* < .001, η_G_^2^ = .016, and Voiceness, *F*(1, 17) = 45.59, *p* < .001, η_G_^2^ = .092, that were opposite to those reported above. The ERP was less positive to surprised and vocal sounds than to neutral and nonvocal sounds (see Fig. 4). Additionally, a Touch × Hemisphere interaction, *F*(2, 34) = 3.76, p_GG_ = .05, η_G_^2^ = .003, indicated that touch modulated the ERP over the left, *F*(2, 34) = 3.92, *p* = .03, η_G_^2^ = .032, but not the right hemisphere (*p* > .250). Over the left hemisphere, we found effects comparable to those described above. The ERP was more positive for arm touch, *F*(1, 17) = 7.1, *p* = .016, η_G_^2^ = .038, and no touch, *F*(1, 17) = 4.89, *p* = .041, η_G_^2^ = .033, as compared with palm touch. Arm touch and no touch did not differ (*p* > .250).

Analysis of the LPP window revealed a Voiceness effect, *F*(1, 17) = 96.24, *p* < .001, η_G_^2^ = .101, and an interaction of Emotion, Voiceness, and Touch, *F*(2, 34) = 4.07, *p* = .026, η_G_^2^ = .005, both pointing to a partial polarity reversal of frontocentral effects. Follow-up analyses showed that the Emotion × Voiceness interaction was significant for arm touch, *F*(1, 17) = 11.05, *p* = .004, η_G_^2^ = .024, but not for palm touch and no touch (*p*s > .250). For arm touch only, the ERP was less positive for surprised as compared to neutral nonvocal sounds, *F*(1, 17) = 9.22, *p* = .007, η_G_^2^ = .05. A similar effect was nonsignificant for vocal sounds (*p* > .250). For palm touch and no touch, the Voiceness main effect indicated that the ERP was less positive for vocal as compared to nonvocal sounds: palm, *F*(1, 17) = 21.21, *p* < .001, η_G_^2^ = .098; none, *F*(1, 17) = 30.89, *p* < .001, η_G_^2^ = .104). The Emotion effect was nonsignificant (*p*s > .250).

## Discussion

Here we explored whether and how touch modulates vocal-emotional processing. We found that touch to both the arm and the palm was perceived as pleasant without changing the participants’ affective state. Moreover, touch did not interfere with the participants’ ability to detect sound repetitions in the experimental task. Yet it modulated how sounds were represented in the brain, and this modulation differed as a function of whether and where participants were touched. What follows is a more detailed discussion of these findings focusing on the nature of vocal-emotional ERP effects, the role of touch for voice perception, and the relevance of our findings for the social brain.

It is well established that the human brain prioritizes emotional over mundane and social over nonsocial information (Schirmer & Adolphs, 2017). In the context of fMRI, affective stimuli excite aspects of the sensory system more strongly than do neutral stimuli (e.g., Grandjean et al., 2005). Similar effects emerge for social as compared to nonsocial stimuli, as in the contrast of faces with nonface objects (e.g., houses; Kanwisher, McDermott, & Chun, 1997), voices with nonvocal sounds (e.g., nature sounds; Belin, Zatorre, Lafaille, Ahad, & Pike, 2000), or touch with human as compared to nonhuman characteristics (e.g., skin temperature; Ackerley et al., 2014; for a review, see Schirmer & Adolphs, 2017). In the ERP, these results are mirrored by greater amplitudes for emotional and social events implying that more neurons are recruited or that neurons respond more synchronously or vigorously (e.g., Bentin, Allison, Puce, Perez, & McCarthy, 1996; Schirmer & Gunter, 2017).

The present results agree with this when considering both the P2 complex and the LPP. In line with existing evidence (Jiang & Pell, 2015; Sauter & Eimer, 2010; Schirmer, Chen, et al., 2013; Schirmer & Gunter, 2017), the P2 complex was larger for emotional relative to neutral sounds over anterior, and marginally so over central regions. Likewise, the P2 was more positive for vocal than for nonvocal sounds over frontocentral regions. Both, the P2 emotion and the P2 voiceness effect were fairly independent of each other and reversed polarity over the mastoids, pointing to possible sources in auditory cortex (Näätänen et al., 2007). Looking at the LPP, we found that emotion and voiceness interacted. A greater LPP emotion effect for vocal as compared to nonvocal sounds implied that affective and social information now became integrated. In other words, listeners, instead of representing emotional significance and humanness separately, now perceived the sound as originating from a surprised human and prioritized the sound in awareness (Schirmer & Gunter, 2017). The frontocentral topography of this process accords with a possible contribution of prefrontal cortex and insula, both brain regions where the different senses as well as higher order perceptual and cognitive processes merge (Klasen, Chen, & Mathiak, 2012; Nieuwenhuys, 2012; Schirmer, Meck, & Penney, 2016) and where emotion effects in the LPP are at least partially regulated (Hajcak et al., 2010; Liu et al., 2012; Moratti, Saugar, & Strange, 2011; Schindler & Kissler, 2016). The LPP mastoid effects, furthermore, raise the possibility that these higher order integrative mechanisms may partially modulate processing in auditory cortex in a top-down manner.

Although the present results inform about vocal-emotional processing, of primary importance are first insights into the role of touch. Such insights emerged by exploring auditory perception in the context of CT-targeted touch to the arm, touch to the palm, and no touch. As expected, we found original evidence for online touch effects that dissociate from simple changes in pleasure or mood. Arm-touch and palm-touch conditions were perceived as equally pleasant, and neither produced subjective mood differences relative to the no-touch control. Yet the two touch conditions distinctly modulated the auditory ERP.

In the P2 time range, palm touch but not arm touch significantly reduced the ERP relative to no touch, suggesting that palm touch in particular interfered with early perceptual representations. Compared to the arm, the palm has a denser distribution of Aβ mechanoreceptors and is hence more sensitive and discriminative (Weinstein, 1968). In the LPP time range, both palm touch and arm touch reduced component amplitudes. However, effects differentiated as a function of emotion and voiceness. Specifically, an interaction between emotion and voiceness showed only anteriorly when participants were stroked on the palm. In contrast, when participants were stroked on the arm, the interaction showed across the scalp. In other words, the larger LPP emotion response to vocal when compared with nonvocal sounds was enhanced for arm touch relative to palm touch. Moreover, LPP effects in the no-touch condition differed from arm touch but not palm touch. Thus, one may speculate that arm touch facilitated the integration of emotional and vocal information and enhanced attentional engagement with emotional voices relative to neutral voices and nonvocal sounds.

The fact that CT afferents are present in the arm but not the palm marks CT afferents as a possible candidate in the present results and suggests that CT stimulation could benefit social interactions by emphasizing socioemotional information. This possibility agrees with existing work on the processing characteristics of CT afferents (for reviews, see Björnsdotter et al., 2010; Schirmer & Adolphs, 2017). Additionally, it accords with other research on touch that did not specifically explore CT effects and that provides more general behavioral and brain evidence for touch effects in adults and children. In adults, a number of field and laboratory studies demonstrated that casual touch increases good will and compliance. In the 1970s, Fisher and colleagues instructed library clerks to return library cards to customers by either briefly touching or not touching them (Fisher et al., 1976). Compared to customers who were not touched, those who were touched rated the library and its staff more positively. Follow-up studies replicated and extended these results showing, for example, that touch increases how much people tip, how honest they are, and whether they take prescribed medication (Schirmer, Wijaya, & Liu, 2016, for a review). More recent evidence from neuroscience complements these findings. In an ERP setting, gentle pressure to the arm, irrespective of whether provided by and attributed to a friend or a machine, enhanced LPP differences between emotional and neutral images that contained social elements (i.e., human or nonhuman animal), implying better emotion differentiation (Schirmer et al., 2011).

Further relevant for the present effects is evidence on the role of parental touch for early social development. A large body of literature documents benefits of skin-to-skin contact during infancy and early childhood (Field, Diego, Hernandez-Reif, Deeds, & Figuereido, 2006; Vickers, Ohlsson, Lacy, & Horsley, 2004), including aspects of social functioning such as the management of negative emotions and responsiveness to caregivers (for a review, see Field, Diego, & Hernandez-Reif, 2010). Children receiving more maternal touch reach out to their mothers more and have an accelerated development of the adult face bias whereby attention shifts to face rather than nonface objects (Reece, Ebstein, Cheng, Ng, & Schirmer, 2016). Furthermore, high-touch children differ from low-touch children in how they activate the “social brain.” When wakefully at rest, they engage the right posterior superior temporal sulcus more strongly and show greater functional connectivity between this region and the medial prefrontal cortex (Brauer, Xiao, Poulain, Friederici, & Schirmer, 2016)—both areas implicated in understanding others’ emotions (Escoffier, Zhong, Schirmer, & Qiu, 2013; Schirmer & Adolphs, 2017). Converging evidence from nonhuman animals implies that tactile stimulation of hairy skin is causally relevant for improved socioemotional functioning in offspring (Zhang & Meaney, 2010).

Although the present study provides compelling insights into the role of touch for neural responses to vocal emotions, it raises a number of questions for future research. First, one may ask why touch effects were absent from the behavioral measures and relatively small in the ERP. Several aspects of experimental design and touch context may be relevant here. For example, an influence on *n*-back performance may become apparent with a larger proportion of response trials. Presently, this proportion was small (16.5 response vs. 81 nonresponse trials per cell in the design) as to maximize the number of nonresponse trials for the ERP (Luck, 2005). In subsequent studies, the proportion of response trials could be increased in combination with an analysis of response-locked ERP components, such as the readiness potential (e.g., Eder, Leuthold, Rothermund, & Schweinberger, 2012). Additional factors that may be relevant for the present touch effects concern the nature of the task and the tactile stimulus. The task directed attention to stimulus acoustics rather than to socioemotional features, thus emphasizing stimulus-driven implicit processing. Future studies may wish to contrast this with explicit processing by engaging participants at a socioemotional level—a condition known to enhance the kind of integrative mechanisms that were of interest here (e.g., Schirmer et al., 2006). Like the task, the tactile stimulus was nonsocial (i.e., brush rather than hand strokes) and applied in a, perhaps artificially, rhythmic and continuous manner, fatiguing CT afferents to some degree (Nordin, 1990) and potentially producing autonomic or hormonal effects (Ditzen et al., 2007; Morhenn, Beavin, & Zak, 2012; Okabe, Yoshida, Takayanagi, & Onaka, 2015). These issues represent the flip-side of our attempt to standardize the physical properties of touch (e.g., touch temperature) and to prevent carryover effects between touch conditions. Future research should use actual human touch as to explore a possible facilitation of touch effects through attributional mechanisms (but see Schirmer et al., 2011). Additionally, a random presentation of touch conditions, together with a long intertrial interval, may enhance touch effects, as short occasional touches may be expected to be more powerful (Triscoli et al., 2014).

A second line of inquiry arising from our results concerns the differential effects of arm and palm touch. On one hand, one may wish to better specify the role of CT afferents. Other receptors differ between nonglabrous and glabrous skin (e.g., hair follicles), offering an alternative explanation for arm/palm differences—an explanation that could be ruled out by, for example, studying certain somatosensory patients (Olausson et al., 2002). On the other hand, one should explore potential commonalities in the touch effects on arm and palm. The fact that palm effects fell somewhere in between arm-touch and no-touch effects suggests that touch, irrespective of location, might shape socioemotional responding. This agrees with human evidence that short touches to glabrous skin impact affect and attitudes (Fisher et al., 1976). It is also in line with evidence from nonhuman animals that have no CT afferents but nevertheless experience stress relief from touch (Schirmer, Jesuthasan, & Mathuru, 2013). Possibly touch provides a very basic signal of being connected with others.

Last, we examined only women here, leaving open the question of whether touch effects differ between the sexes and between same and opposite sex interactions. Extant research points to such effects. Touch studies that addressed differences between female and male touchees found a greater response in the former as compared with the latter group (Fisher et al., 1976; Patterson, Powell, & Lenihan, 1986). In line with this, there is evidence for greater female sensitivity to facial (e.g., Schirmer, Seow, & Penney, 2013) and vocal expressions (e.g., Schirmer & Gunter, 2017)—especially when expressions are subtle or task-irrelevant. Additionally, how men and women direct their attention is modulated by the sex of interaction partners in complex ways (Alexander & Charles, 2009; Amon, 2015). In the context of touch, biases have been identified (e.g., young men touch young women more than vice versa; Hall, 1996; Hall & Veccia, 1990) that need to be explored vis-à-vis their perceptual consequences. In fact, the interplay of sensory (i.e., tactile stimulus) and attributional (i.e., who is touching whom) effects in how the sexes experience touch constitutes an interesting avenue for further research, with potential applications to clinical disorders that are marked by sex-specific prevalences (e.g., autism and depression).

To conclude, this study revealed distinct influences of arm touch and palm touch on ongoing mental functioning. Palm touch interfered with cross-modal perceptual and attentional processing as indicated by a reduction of the auditory ERP in both P2 and LPP time range. In contrast, arm touch affected the LPP only and did so in a more differentiated manner. Specifically, it facilitated the integration of vocal and emotional information and dampened responses to sounds, excepting those to emotional voices. Together, these results point to the activation of CT afferents in hairy skin as a possible mechanism for the facilitation of neural responses to social signals with affective relevance. Such stimulus-driven effects may then interact with the influence of contextual factors (e.g., social goals, toucher–touchee relationship) to shape social attitudes and behavior. Thus, in interactional settings as diverse as a mother caring for her child or business colleagues negotiating a contract, social touch may facilitate emotional exchange.

## Electronic supplementary material

ESM 1 (DOC 133 kb)
