# Supplementary material for: The right touch: Stroking of CT-innervated skin promotes vocal emotion processing
Source: Cogn Affect Behav Neurosci. 2017 Sep 20;17(6):1129–40. doi: 10.3758/s13415-017-0537-5 (PMC5709431; doi:10.3758/s13415-017-0537-5)
Supplement: Supplementary file 1 — (DOC 133 kb) [file 13415_2017_537_MOESM1_ESM.doc]

**Supplementary Results**

Analysis of data referenced to the average of all scalp electrodes.


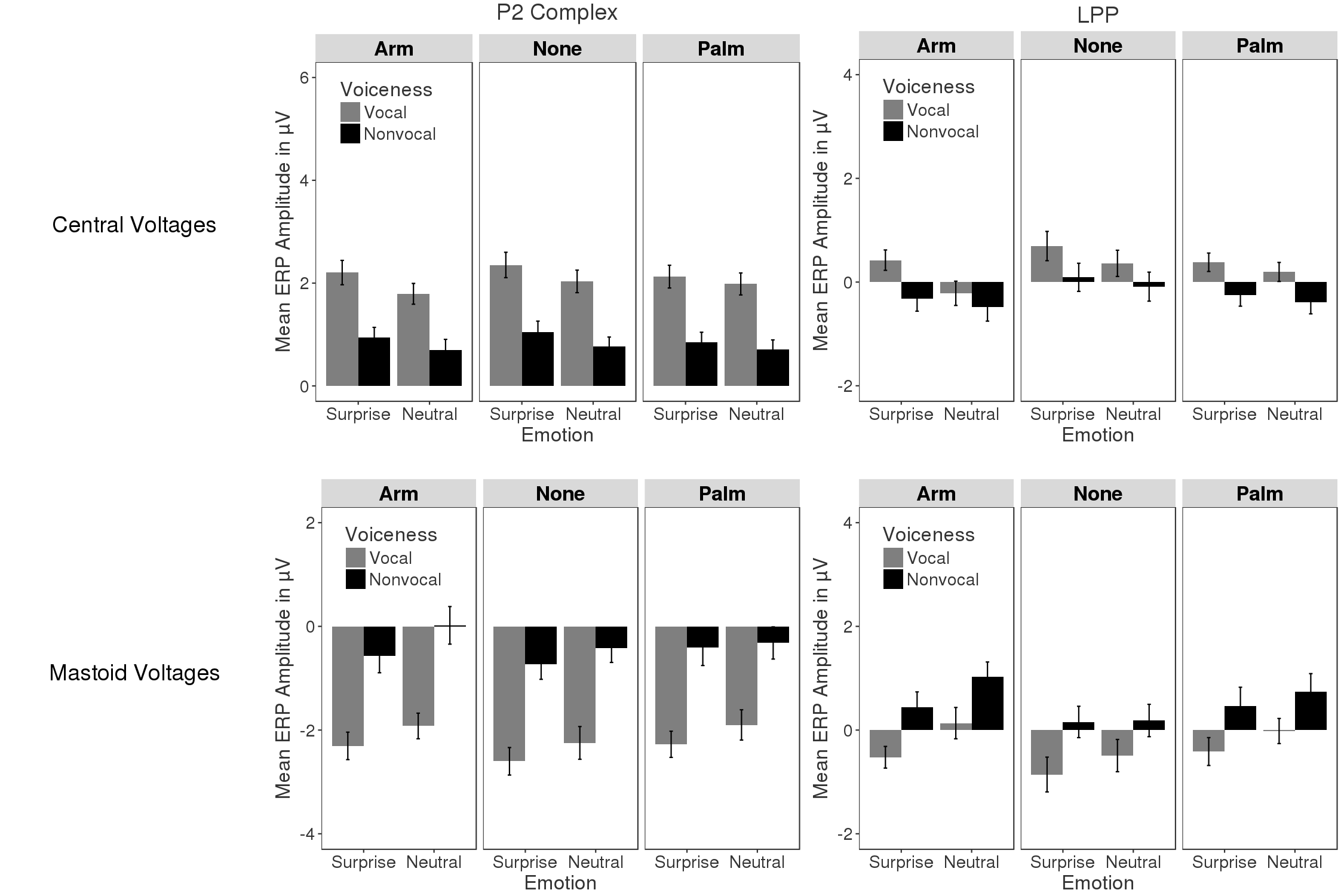


Supplementary figure. Bargraph of P2 (left) and LPP (right) mean voltages for the different experimental conditions computed across the left and right central (top) and mastoid (bottom) region. Voiceness effects are most evident in the P2 with greater amplitude for vocal as compared with nonvocal sounds. Touch effects are most evident in the LPP where they interact with voiceness and emotion. Error bars reflect the 95% confidence interval.

**P2 Analysis**

| ***Effect*** | ***DF*** | ***F*** | ***P*** | ***GES*** |
| --- | --- | --- | --- | --- |
| Emotion | 1,17 | 11.69 | <.0001 | 0.0013 |
| Voiceness | 1,17 | 100.44 | <.0001 | 0.032 |
| Emotion*Region | 2,34 | 36.9 | <.0001 | 0.013 |
| Voiceness*Region | 2,34 | 61.38 | <.0001 | 0.079 |
| Emotion*Voiceness*Region | 2,34 | 4.62 | 0.017 | 0.0019 |
| Emotion*Hemisphere*Region | 2,34 | 8.25 | 0.001 | 0.0002 |
| Voiceness*Hemisphere*Region | 2,34 | 7.93 | 0.001 | 0.001 |

Other ps > .101

**LPP Analysis**

| ***Effect*** | ***DF*** | ***F*** | ***P*** | ***GES*** |
| --- | --- | --- | --- | --- |
| Touch | 2,34 | 3.41 | 0.045 | 0.0022 |
| Emotion | 1,17 | 21.9 | 0.0002 | 0.0013 |
| Voiceness | 1,17 | 45.09 | <0.0001 | 0.0104 |
| Emotion*Voiceness | 1,17 | 6.92 | 0.017 | 0.0003 |
| Emotion*Region | 2,34 | 5.67 | 0.007 | 0.0026 |
| Voiceness*Region | 2,34 | 48.66 | <0.0001 | 0.0506 |
| Emotion*Hemisphere*Region | 2,34 | 8 | 0.001 | 0.0002 |
| Voiceness*Hemisphere*Region | 2,34 | 3.52 | 0.041 | 0.0004 |
| Touch*Emotion*Voiceness*Hemisphere | 2,34 | 2.95 | 0.066 | 0.0003 |
| Touch*Emotion*Voiceness*Region | 4,68 | 2.61 | 0.043 | 0.0013 |
| Touch*Voiceness*Hemisphere*Region | 4,68 | 2.11 | 0.088 | 0.0001 |

Other ps > .136
